# Stem cell extracellular vesicles: a new dawn for anti-inflammatory treatment of neurodegenerative diseases

**DOI:** 10.3389/fnagi.2025.1592578

**Published:** 2025-07-11

**Authors:** Miao Yu, Hongxia Ma, Xiaoyi Lai, Jiannan Wu, Mengmeng Shen, Junqiang Yan

**Affiliations:** ^1^Key Laboratory of Neuromolecular Biology, The First Affiliated Hospital of Henan University of Science and Technology, Luoyang, China; ^2^Department of Neurology, The First Affiliated Hospital, College of Clinical Medicine of Henan University of Science and Technology, Luoyang, China

**Keywords:** stem cell therapy, extracellular vesicles, neuroinflammation, neurodegenerative diseases, Alzheimer’s disease, Parkinson’s disease, amyotrophic lateral sclerosis, multiple sclerosis

## Abstract

Mesenchymal stem cell-derived extracellular vesicles, as carriers for intercellular communication, are rich in bioactive substances such as proteins and nucleic acids, and show unique potential in the treatment of neurodegenerative diseases. Their vesicular structure, with a diameter of 30–150 nm, can penetrate the blood–brain barrier and modulate the activity of microglia and astrocytes by delivering functional molecules. This process inhibits the release of pro-inflammatory factors and enhances the expression of anti-inflammatory mediators, thereby alleviating neuroinflammation in the pathological process of neurodegenerative diseases. As natural drug carriers, extracellular vesicles can improve the targeted delivery efficiency of therapeutic molecules. However, their specific anti-inflammatory mechanisms remain not fully understood and require further exploration. This article discusses the anti-inflammatory effects in the context of neurodegenerative diseases and provides a summary and outlook on the anti-inflammatory actions associated with extracellular vesicles from past research.

## 1 Introduction

Stem cells possess unique properties that enable them to differentiate into various cell types and self-renew *in vivo* (Zakrzewski et al., 2019). Extracellular vesicles (EVs) facilitate intercellular communication by carrying various biologically active molecules, including proteins, lipids, and RNA. Mesenchymal stem cell-derived extracellular vesicles (MSC-EVs) have been shown to regulate nerve-related cell functions, reduce neuroinflammation, and thus contribute to neuroprotection. EVs can affect the expression of genes related to inflammation and apoptosis, thereby promoting neuronal survival and reducing inflammatory response (Zhang et al., 2023). MSC-EVs enhance angiogenesis, essential for neurodegenerative disease (NDD) recovery, through upregulation of intercellular cell adhesion molecule-1 (ICAM-1) and activation of associated signaling pathways (Xue et al., 2021). Improving blood flow helps reduce inflammation and supports neuronal health. On the other hand, MSC secrete or package neurotrophic factors into EVs or exosomes, which can promote angiogenesis and have a protective effect on dopaminergic neurons (d’Angelo et al., 2020; Hofer and Tuan, 2016). More importantly, EVs are rich in miRNA, which can regulate gene expression in recipient cells. The miR-100-5p found in MSC-EVs derived from the trophoblast stage has been shown to target NADPH oxidase 4 (NOX4). By inhibiting NOX4 in a Parkinson’s disease (PD) model, these EVs can reduce the production of reactive oxygen species, thereby mitigating oxidative stress and its inflammatory consequences in dopaminergic neurons (He et al., 2023). The ability of EVs to cross the blood–brain barrier (BBB) enhances their therapeutic potential by allowing them to exert anti-inflammatory effects directly within the central nervous system (CNS) (Chen et al., 2020). The use of EVs as a treatment strategy has many advantages over traditional stem cell therapy. Compared with whole-cell therapy, EVs can be produced in large quantities and have a lower risk of tumorigenicity and immune rejection. This makes them a more feasible choice for clinical application in the treatment of NDDs.

Neurodegenerative diseases are a group of diseases caused by the gradual loss of specific neuronal populations, affecting the lives of millions of people around the world (Heemels, 2016). Neurodegeneration is the gradual loss of neuronal structure and function, including neuronal death and glial cell imbalance, which can lead to cognitive impairments such as dementia. Currently, common NDDs include Alzheimer’s disease (AD), PD, amyotrophic lateral sclerosis (ALS), and multiple sclerosis (MS). As the global population ages, the incidence of NDDs is increasing annually, placing a substantial burden on patients’ families and society. The pathogenesis of these diseases is complex, often involving multiple pathological processes such as neuroinflammation, oxidative stress, and apoptosis. Recent studies have demonstrated that inflammation plays a crucial role in disease progression, suggesting that inhibiting neuroinflammation may offer new therapeutic strategies. The inflammatory response is mainly driven by glial cells such as microglia and astrocytes, rather than by infiltration of peripheral white blood cells (Minghetti, 2005). The regulation of inflammation relies on the ability of microglia and macrophages to exhibit two opposing phenotypes: the M1 phenotype, which can produce cytotoxic effects, and the M2 phenotype, which can produce neuroprotective effects (Amici et al., 2017). The dynamic changes in M1/M2 phenotypes are closely associated with NDDs, including AD, PD, ALS, and MS (Tang and Le, 2016). The phenotype of inflammatory cells changes depending on the stage and severity of the disease. Furthermore, effectively managing the phase transition of the M1/M2 phenotypes within an appropriate time window may enhance therapeutic outcomes (Carata et al., 2023). In some cases, the inflammatory response can promote the recovery of damaged neurons, but it may also lead to or aggravate NDDs. Studies have shown that although acute inflammation may help repair damaged neurons, persistent chronic inflammation often leads to neuronal dysfunction and death (Wyss-Coray and Mucke, 2002). Inflammatory factors can regulate neuronal excitability and synaptic function by interacting with specific receptors on the surface of neurons. In NDDs, inflammation is typically triggered by the accumulation of abnormal proteins, such as amyloid-beta and tau, or signals released by damaged cells. These abnormal proteins and cell signals activate microglia, leading to the inflammatory response (Ising et al., 2019; Kwon and Koh, 2020; Leng and Edison, 2021; Peterson and Fujinami, 2007; Wang et al., 2015). It is particularly important to emphasize that the traditional microglia M1/M2 dichotomy has been proven insufficient to fully reflect their highly dynamic and heterogeneous functional states. Recent single-cell transcriptomics studies have revealed that microglia undergo continuous functional state transitions in NDDs, involving multi-dimensional features such as metabolic reprogramming, phagocytic functional plasticity, and microenvironment-dependent epigenetic remodeling (Paolicelli et al., 2022). This will provide new theoretical foundations for the dynamic impact of precise therapeutic strategies using stem cell-derived EVs, while simultaneously emphasizing the heterogeneity of microenvironment-specific regulatory targets at different stages of disease progression.

## 2 Characteristics of stem cell extracellular vesicles

### 2.1 Classification and function of stem cells

Mesenchymal stem cells are a type of stem cell with multidirectional differentiation potential, naturally found in tissues such as bone marrow, adipose tissue, and umbilical cord (Galipeau and Sensébé, 2018). They can be isolated and expanded using adherent culture methods. Their ability to differentiate into CNS cells depends on specific induction conditions, such as chemical factors like β-mercaptoethanol and retinoic acid or regulation by growth factors like brain-derived neurotrophic factor (BDNF) (Liu et al., 2022a). iPSCs are pluripotent stem cells reprogrammed from mature somatic cells (Malik and Rao, 2013). This reprogramming is achieved by introducing key transcription factors such as octamer-binding transcription factor (OCT4), sex determining region Y-box 2 (SOX2), Krüppel-like factor 4 (KLF4), and c-Myc into mature somatic cells, like skin fibroblasts. Subsequently, these cells can be directed to differentiate into neural precursor cells by inhibiting the SMAD signaling pathway. Further combination with developmental signals such as FGF8 can induce differentiation into specific CNS cell types, such as dopaminergic neurons (Huang et al., 2024). Furthermore, three-dimensional organoids or microglial cells (iMGLs) derived from iPSCs can be used to study NDDs by simulating the microenvironment of brain development (Bhargava et al., 2022; Karagiannis et al., 2019). The advantages of MSCs lie in their inherent immunomodulatory functions and ease of acquisition, while iPSCs can construct patient-specific disease models. Both have complementary potential in exploring neural regeneration mechanisms and clinical translation (Joo et al., 2020; Shi et al., 2024).

### 2.2 Structure and biological function of extracellular vesicles

#### 2.2.1 The formation and secretion process of extracellular vesicles

**FIGURE 1:**
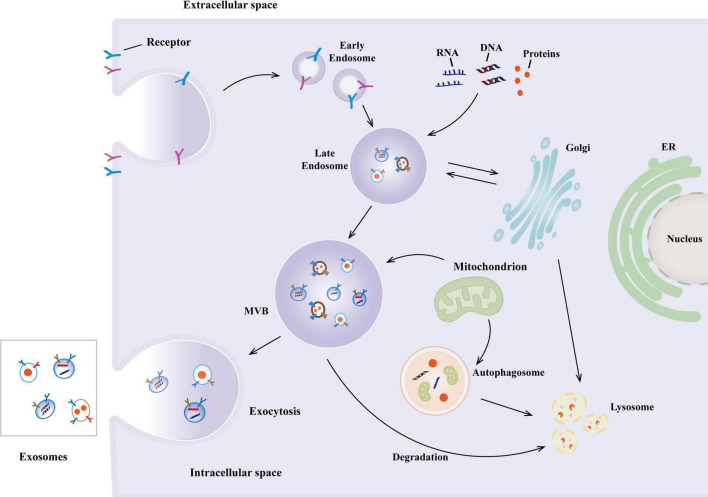
The biogenesis of exosomes. The biogenesis of exosomes begins with the formation of early endosomes in the endocytosis of the cell membrane and subsequently matures into multivesicular bodies (MVBs), in which intraluminal vesicles (ILVs) are formed through membrane invagination. After MVBs fuse with the plasma membrane, ILVs are released as exosomes.

Extracellular vesicles were first discovered in 1981, initially known as exfoliative vesicles (Trams et al., 1981). EVs, are lipid-delimited biological nanoparticles that appear to be released by all cell types. EVs are usually between 30 and 150 nm in diameter. The biogenesis of exosomes begins with the endocytosis of the cell membrane forming early endosomes (Wang et al., 2024; Xie et al., 2022) and subsequently matures into multivesicular bodies (MVBs), within which intraluminal vesicles (ILVs) are formed through membrane invagination (Hessvik and Llorente, 2018; Yue et al., 2020). After MVBs fuse with the plasma membrane, ILVs are released as exosomes, a process dependent on the Rab protein family and membrane fusion mechanisms (Kim et al., 2018; Xu et al., 2023) (Figure 1). In addition to exosomes that originate from the endocytic pathway, cells can also secrete small extracellular vesicles (sEVs) directly through budding from the plasma membrane. Their formation involves the ESCRT complex or lipid raft microdomains (Gurung et al., 2021; Ishii et al., 2023; Moyano et al., 2019). Recent studies emphasize the high heterogeneity of EVs, including subtypes such as exosomes, microvesicles, and apoptotic bodies, which exhibit significant differences in biogenetic pathways and molecular characteristics (Rincón-Riveros et al., 2021; Yuan et al., 2023). Their efficient delivery capability may be related to their natural or acquired tropism, which allows them to cross cellular barriers and result in fewer off-target effects compared to synthetic nanoparticles (Elsharkasy et al., 2020; Witwer and Wolfram, 2021). EVs derived from MSCs can modulate signaling pathways in recipient cells, playing important roles in tissue repair and immune regulation (Gurung et al., 2021; Yu et al., 2020; Yuan et al., 2023). Recent advancements demonstrate that engineering modifications of EV surface proteins can enhance their therapeutic specificity, such as the simultaneous display of two decoy receptors significantly improving anti-inflammatory effects. These findings highlight the unique advantages of EVs as natural delivery vectors (Cheng and Hill, 2022; Gupta et al., 2021; Herrmann et al., 2021; Kalluri and LeBleu, 2020; Möller and Lobb, 2020; van Dommelen et al., 2012).

#### 2.2.2 Bioactive components of extracellular vesicles

Extracellular vesicles contain a range of proteins, including membrane and cytoplasmic proteins, which are crucial for cell signal transduction, intercellular interactions, and immune responses (Yuan et al., 2023). The quantity of bioactive molecules transported by EVs varies depending on the type of cell (Jeppesen et al., 2019) and the stage of cell differentiation (Romancino et al., 2013). MSC-EVs are typically rich in growth factors and cytokines related to cell survival, proliferation, and differentiation, such as transforming growth factor (TGF) and platelet-derived growth factor (PDGF) (Tan et al., 2024). The lipid bilayer membrane of EVs not only maintains structural integrity but also carries various bioactive substances that are essential for regulating their biological functions (Sun et al., 2021). EVs contain various effector proteins, messenger RNA (mRNA), microRNA (miRNA), and mitochondrial RNA (mtRNA), mitochondrial DNA (mtDNA), which can potentially affect the fate of target cells by regulating gene expression through interaction with receiving cells (Fakouri et al., 2024).

## 3 The anti-inflammatory mechanism of stem cell extracellular vesicles

### 3.1 The role of extracellular vesicles in immune regulation

Immune cell-derived exosomes (IEXs) are key mediators of intercellular communication that play a central, bidirectional regulatory role in inflammatory responses, with their functions being highly dependent on the type and state of their parent cells (Glass et al., 2010; Hickman et al., 2018). When activated, microglia exhibit heterogeneous responses toward surrounding cells, manifesting either pro- or anti-inflammatory effects (Ajami et al., 2018; Ransohoff, 2016). Microglia-driven inflammation may further compromise astrocytic function, impairing their capacity to support neuronal survival and growth while promoting neurotoxic phenotypes (Jha et al., 2019; Liddelow et al., 2017). Under pathological conditions such as hypertension, activated macrophages release EVs enriched with pro-inflammatory factors. Concurrently, reduced levels of miR-17—a negative regulator of ICAM-1—in these EVs establish a pro-inflammatory feedback loop that activates the downstream NLRP3 inflammasome pathway, thereby amplifying the inflammatory cascade (Hazrati et al., 2022; Osada-Oka et al., 2017). Conversely, MSC-EVs demonstrate potent anti-inflammatory and immunomodulatory properties. These EVs induce regulatory T cell (Treg) differentiation, suppress pro-inflammatory Th17 cell activity (Heidari et al., 2022; Yang et al., 2021), and modulate dendritic cell (DC) signaling pathways through targeted molecular delivery. MSC-EVs engineered to overexpress miR-540-3p inhibit the CD74/NF-κB axis in DCs, reducing pro-inflammatory cytokines IL-1β, IFN-γ while enhancing anti-inflammatory mediators IL-10, TGF-β1, ultimately attenuating inflammation and tissue damage (He et al., 2024). Similarly, Treg-derived EVs deliver bioactive molecules (dimethyl fumarate, DMF) directly to local tissue cells such as keratinocytes, suppressing inflammation and promoting immune tolerance (Ou et al., 2025). Collectively, stem cell EVs function as versatile carriers of specific biomolecular “cargo,” possessing inherent cell-targeting capabilities, engineerable plasticity for functional reprogramming, and context-dependent duality in disease pathogenesis.

### 3.2 Inhibition of proinflammatory cytokines by extracellular vesicles

Mesenchymal stem cell-derived extracellular vesicles exert immunomodulatory effects by regulating the function of immune cells. In a lipopolysaccharide-induced neuroinflammation mouse model, MSC-EVs can inhibit microglial activation and significantly reduce the levels of pro-inflammatory factors TNF-a and IL-6 in the hippocampal region (Domenis et al., 2018). This anti-inflammatory effect is particularly prominent in models of chronic NDDs. For instance, studies on transgenic mice with AD have shown that EVs treatment can reduce inflammatory infiltration around amyloid plaques (Wang et al., 2021). Notably, specific cytokines can optimize the therapeutic potential of EVs. Croitoru-Lamoury et al. (2011) discovered that IFN-γ-stimulated bone marrow MSCs enhance immunosuppressive capabilities by upregulating indoleamine-2,3-dioxygenase (IDO) expression, a mechanism that has shown efficacy in experimental autoimmune encephalomyelitis (EAE) mouse models. MSC-EVs may exert their effects by carrying specific microRNAs. In the serum EVs model of cynoglossus semilaevis, Sun et al. (2022) found that miR-133-3p can reduce the production of IL-1β and IL-6 by inhibiting the PP2A/NF-κB pathway, a mechanism that has also been validated in mammalian macrophage lines. MSC-EVs treated with proinflammatory cytokines have been found to enhance immunosuppressive and anti-inflammatory potential (Domenis et al., 2018). Wozniak et al. (2020) demonstrated, using a hepatitis virus infection model, that inflammatory signals can promote the selective loading of exosomal miRNA mediated by the RNA-binding protein FMR1. Additionally, EVs can reduce pro-inflammatory responses by inhibiting the formation of inflammasome complexes. The systematic review by Noonin and Thongboonkerd (2021) indicates that MSC-EVs can inhibit caspase-1 activation and IL-1β maturation in a sepsis model by blocking the assembly of the NLRP3 inflammasome. These studies suggest that EVs have different regulatory mechanisms in various stages of inflammation and disease models.

### 3.3 Extracellular vesicles and intercellular communication

This communication is crucial for coordinating cellular responses and maintaining tissue homeostasis (Hade et al., 2021; Tan et al., 2024). EVs can transfer bioactive molecules from one cell to another. This process can be carried out through direct cell contact or through intercellular space. When EVs bind to target cells, the molecules in EVs can be released and affect the function of target cells. MiRNAs in stem cell EVs can regulate gene expression in target cells, thereby affecting cell proliferation and differentiation (Liu et al., 2022b; Singh G. et al., 2024). MiRNAs play a crucial role in regulating the development and function of DCs. Studies have shown that miRNAs can influence the maturation, migration, and antigen presentation of DCs, thereby affecting the strength and nature of the immune response (Zhou and Wu, 2017). MiRNAs not only regulate gene expression independently, but also regulate the function of peripheral cells by interacting with signal transduction pathways. In response to peripheral nerve injury, the expression pattern of miRNAs changes and regulates signaling pathways related to nerve protection and regeneration, suggesting that miRNAs play a crucial role in the repair and regeneration of peripheral nerves (Borger et al., 2022).

## 4 The core pathogenic role of inflammation in neurodegenerative diseases

### 4.1 Alzheimer’s disease

Alzheimer’s disease is a chronic, progressive neurodegenerative disorder, accounting for approximately 60%–80% of all dementia cases (De-Paula et al., 2012; Scheltens et al., 2021). It is characterized by cognitive and behavioral impairments that significantly affect patients’ social and occupational abilities (Zhang et al., 2024). Currently, it is an incurable disease with a lengthy preclinical course. The pathogenesis of AD is not yet fully understood. Its main characteristics involve the synergistic pathological interaction between amyloid beta (Aβ) accumulation and excessive phosphorylation of tau protein, both of which are key drivers of neurodegenerative lesions (Burns and Wang, 2017; Ising et al., 2019; Madar et al., 2024). The study found that microglia are activated in response to amyloid plaques, leading to the release of pro-inflammatory cytokines that contribute to neuroinflammation and neuronal damage (Balducci and Forloni, 2018; Leng and Edison, 2021; von Bernhardi et al., 2015).

In summary, Aβ plaques induce synaptic dysfunction through amyloid-mediated interference and further trigger cascade events that directly promote tau pathology (Abdelnour et al., 2022; Hyman et al., 2012; Serrano-Pozo et al., 2011). Research indicates that Aβ oligomers activate the NLRP3 inflammasome in microglia, promoting the release of interleukin-1β (IL-1β), thereby enhancing tau kinase activities (such as GSK-3β) while inhibiting phosphatases. This neuroinflammatory environment leads to excessive tau phosphorylation, tangle formation, and subsequent neuronal death (Cummings, 2021; Wang C. et al., 2022; Zhao et al., 2024). In mouse models, intracerebral injection of Aβ induces tau pathology, while NLRP3 gene knockout alleviates tau aggregation and cognitive deficits (Wang C. et al., 2022; Zhao et al., 2024). This creates a self-amplifying cycle in which Aβ-induced neuroinflammation exacerbates tau toxicity, ultimately accelerating synaptic loss and hippocampal atrophy (Pini et al., 2016; Singh M. et al., 2024; Tangaro et al., 2014). It has been found that the release of TNF-a, IL-1β, and nitric oxide (NO) regulates the transformation of microglia into pro-inflammatory phenotype (Huang R. et al., 2023; Li et al., 2023). A recent study demonstrated that C-X-C motif chemokine ligand 10 (CXCL10) and its receptor, CXCR3, are pivotal in regulating the infiltration of CD8+ T cells into the brain. These infiltrating CD8+ T cells interact with microglia, collectively promoting neurodegenerative changes associated with AD (Jorfi et al., 2023). TREM2 is a potential therapeutic target due to its neuroprotective role in early AD, mitigating neuroinflammation, cognitive impairment, and M1 microglia polarization via the PI3K/AKT/FoxO3a pathway in AD models (Wang Y. et al., 2020).

**FIGURE 2:**
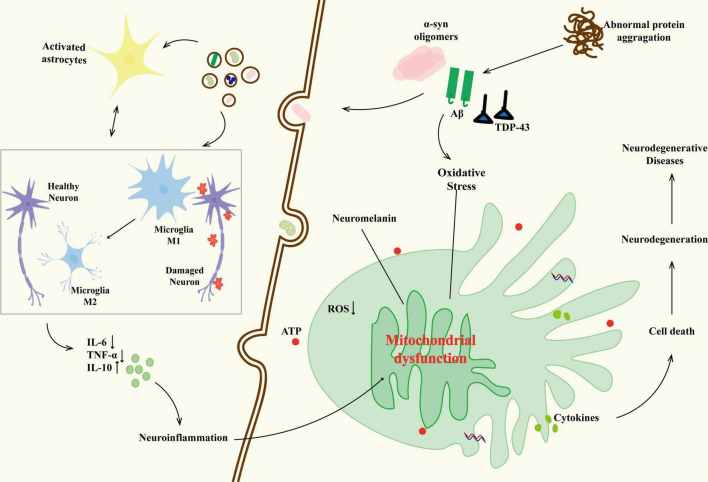
The relationship between neurodegenerative diseases and glial cells. The abnormal protein aggregation activates microglia (M1 phenotype releases IL-6 and TNF-a) and triggers their interaction with astrocytes (IL-6), forming a positive feedback loop of neuroinflammation. As the disease progresses, some M1 microglia transform into the M2 phenotype, releasing IL-10 in an attempt to repair; however, their function is inhibited by the oxidative stress microenvironment. The persistent activation of glial cells results in neuronal mitochondrial dysfunction (reduced ATP production, oxidative stress imbalance), abnormal deposition of neuromelanin, and loss of synaptic plasticity, ultimately driving the malignant progression of neurodegenerative diseases through blood–brain barrier leakage and inflammatory factor infiltration.

Tau pathology exacerbates Aβ accumulation by impairing microglial clearance functions and promoting astrocyte dysfunction. Reactive astrocytes lose their ability to degrade Aβ (Han et al., 2019; Lana et al., 2023; Rather et al., 2021) and release pro-inflammatory mediators TNF-a, IL-6, further polarizing microglia toward a neurotoxic phenotype (Kitazawa et al., 2005; Novoa et al., 2022). This interaction between glial cells underscores the interdependence of Aβ and tau, as both pathologies jointly sustain chronic neuroinflammation and neuronal death (Arranz and De Strooper, 2019; Gopinath et al., 2023; Rossi and Volterra, 2009). Emerging therapeutic strategies reflect attention to this dual pathology, with MSCs modulating microglial polarization toward an anti-inflammatory phenotype (M2) through the secretion of TGF-β1, resulting in better cognitive recovery compared to single-target approaches (Ju and Tam, 2020; Wang Y. et al., 2020) (Figure 2).

### 4.2 Parkinson’s disease

Parkinson’s disease is the second most common NDDs after AD. Globally, 10 million patients are affected, with a higher incidence among people over 60 years old (Dorsey et al., 2018; GBD 2016 Neurology Collaborators, 2019). The core pathological features are the progressive loss of dopaminergic neurons in the substantia nigra and the formation of Lewy bodies (LBs) (Tolosa et al., 2021; Ye et al., 2023). The death of dopaminergic neurons leads to motor symptoms, tremor, rigidity, and non-motor symptoms, cognitive impairment, mood disorders, directly related to the dysfunction of the nigrostriatal pathway (Chaudhuri et al., 2006; Pfeiffer, 2016; Poewe, 2008). Neuroinflammation is a key driving factor in the progression of PD (Grayson, 2016; MacMahon Copas et al., 2021; Samii et al., 2004). The degree of microglial activation is associated with the dopaminergic terminal loss in early-stage PD (Ouchi et al., 2005). Abnormal aggregation of a-synuclein is not only a major component of LBs but also triggers a neuroinflammatory cascade by activating the TLR4/NLRP3 inflammasome pathway in microglia, leading to the release of pro-inflammatory factors such as IL-1β and IL-18 (Fu et al., 2023). This inflammatory microenvironment exacerbates neuronal damage and motor/cognitive dysfunction by regulating synaptic plasticity and neurotransmitter metabolism (Erta et al., 2012; Santello and Volterra, 2012). Promoting the activation of M2-type microglia may have potential benefits in slowing the progression of PD (Badanjak et al., 2021). The NLRP3 inflammasome is activated by ROS, upregulating IL-18 levels, which is positively correlated with the severity of motor symptoms and can serve as an inflammatory biomarker for PD (Cabrera Ranaldi et al., 2023). There is a bidirectional regulation between the metabolic reprogramming of microglia and the activation of the NLRP3 inflammasome: mitochondrial dysfunction can enhance inflammasome assembly, whereas blocking NLRP3 can reverse the polarization of microglia toward the pro-inflammatory M1 type (Huang R. et al., 2023). Additionally, peripheral inflammation promotes the spread of central inflammation through BBB leakage, and elevated serum CRP in elderly patients is associated with increased risk of PD (Jin et al., 2020). Imaging studies show a positive correlation between the degree of microglial activation in the substantia nigra of PD patients and the loss of dopaminergic terminals, providing a dynamic assessment indicator for inflammation-targeted therapies (Tansey et al., 2022). EVs can act as central conduits for the transmission of a-synuclein, amplifying neuroinflammation and shuttling peripheral inflammatory signals, making them key diagnostic/therapeutic targets.

### 4.3 Amyotrophic lateral sclerosis

Amyotrophic lateral sclerosis is a NDD that primarily affects motor neurons in the brain and spinal cord (Feldman et al., 2022). Known for its progressive and lethal nature, the disease gradually causes patients to lose muscle control, ultimately affecting breathing and other basic physical functions (Hardiman et al., 2017). ALS is pathologically characterized by degeneration of upper motor neurons in the cerebral cortex and lower motor neurons in the spinal cord/brainstem (Rizea et al., 2024; van den Bos et al., 2019). With a global incidence rate of 1–2 per 100,000, this neurodegenerative disorder demonstrates epidemiological patterns of slight male predominance and higher prevalence in middle-aged and elderly populations. Despite being classified as a rare disease, its progressive and debilitating nature imposes substantial physical and psychological burdens on both patients and their families (Arthur et al., 2016; Xu et al., 2020). Its core pathological features include abnormal aggregation of TDP-43 protein within motor neurons and a glial cell-mediated neuroinflammatory cascade (de Boer et al., 2020; Lee et al., 2011). Recent studies emphasize that neuroinflammation driven by non-neuronal cells (Appel et al., 2021; Clarke and Patani, 2020) is a key mechanism accelerating disease progression: the polarization of microglia from an early protective M2 phenotype to a late toxic M1 phenotype (Liao et al., 2012), together with astrocytes forming a pro-inflammatory microenvironment through Cx30-mediated inflammatory signaling (Hashimoto et al., 2022). The degeneration of oligodendrocytes leads to dysfunction, causing axonal lesions in the motor neurons of ALS patients (Kang et al., 2013; Philips et al., 2013). Activation of the NLRP3 inflammasome results in the release of cytokines such as IL-1β and IL-18 (Beers and Appel, 2019), forming an inflammatory amplification loop along with the NF-κB pathway abnormalities induced by optineurin (OPTN) gene mutations (Akizuki et al., 2013; Markovinovic et al., 2018; Toth and Atkin, 2018). Additionally, some ALS cases have a familial hereditary nature. Known genetic mutations, such as superoxide dismutase 1 (SOD1) and C9orf72, are closely related to the pathogenesis of this disease (Marino et al., 2015; Ross and Poirier, 2005). Furthermore, these mutations may affect protein folding and function, leading to neuronal dysfunction and death (Aguzzi and Rajendran, 2009; Polymenidou and Cleveland, 2011). Notably, specific interventions targeting astrocytes in SOD1 mutant mice have confirmed that targeting glial cell inflammation can significantly delay disease progression (Ferraiuolo et al., 2016; Wang et al., 2011). Thus, EVs act as central performers to direct the transport of mutant cargo that drives neuronal protein toxicity, while providing actionable targets for intervention through EV modulation or engineering.

### 4.4 Multiple sclerosis

Multiple sclerosis is a complex, chronic autoimmune disease affecting the CNS (Bjornevik et al., 2022; Olsson et al., 2017), where the immune system mistakenly attacks the myelin sheath of the CNS, leading to neuronal damage and dysfunction. As the myelin is progressively damaged, patients may experience a range of symptoms, including motor dysfunction, sensory disturbances, and visual problems (Correale et al., 2017; Doshi and Chataway, 2016). This immune response may be driven by genetic susceptibility involving variations in multiple immune-related genes (Ghasemi et al., 2017; Goodin, 2024), which synergistically interact with environmental factors [such as Epstein-Barr virus (EBV) infection and vitamin D deficiency] through epigenetic regulation to produce a pathogenic effect (Fujinami et al., 2006; O’Gorman et al., 2014; Speer, 2013; Zhang et al., 2000). The core pathological hallmark manifests as autoimmune-mediated destruction of CNS myelin sheaths, with disease progression being persistently accompanied by continuously evolving neuroinflammatory processes (Compston and Coles, 2008; Lassmann, 2018). Investigations reveal that microglia and astrocytes exhibit spatiotemporal-specific activation patterns in neuroinflammatory cascades: During acute phases, microglia facilitate tissue repair through myelin debris clearance and anti-inflammatory factor secretion (Dendrou et al., 2015; Dobson and Giovannoni, 2019; Lassmann, 2018), whereas chronic activation exacerbates axonal damage via pro-inflammatory mediators, such as TNF-a (Arnett et al., 2001; Liu et al., 1998). Notably, this cytokine demonstrates biphasic regulatory capacity by concurrently promoting oligodendrocyte precursor cell proliferation (Compston and Coles, 2008; Marcus, 2022). Aberrant activation of the receptor-interacting protein kinase 1 (RIPK1) signaling pathway has been identified as a critical molecular mechanism driving neuroinflammation, demonstrating specific upregulation in MS patients’ brain tissues and mediating the formation of astrocyte-microglia inflammatory networks (Zelic et al., 2021). At the immunoregulatory level, anti-inflammatory cytokines IL-4 and IL-10 exert direct neuroprotective functions through modulation of Th cell differentiation (Hulshof et al., 2002; Wang et al., 2018), with their receptors exhibiting specific distribution patterns in MS lesions. While astrocyte-derived glial scars effectively confine inflammatory spread, their physical barrier properties may concurrently impair regenerative processes, this dual functionality shows close association with cellular activation states and microenvironmental factors (Ponath et al., 2018; Sen et al., 2022; Sofroniew and Vinters, 2010; Voss et al., 2012). Furthermore, BDNF has demonstrated pivotal neuroprotective effects in EAE models by sustaining oligodendrocyte survival and promoting myelin regeneration (Linker et al., 2010), whereas IFN-κ’s immunomodulatory functions influence disease progression through Th1/Th2 balance regulation (Billiau et al., 1988).

## 5 The application of stem cell extracellular vesicles in neurodegenerative diseases

### 5.1 *In vitro* studies

Stem cell extracellular vesicles show multidimensional therapeutic potential in *in vitro* models of NDDs. Its core mechanism relies on the inherent biologically active “cargo” delivery capacity of EVs. Neuroprotection, human amniotic fluid stem cell exosomes (hAFSC-Exos) play a crucial role by inhibiting microglial activation. After exosomal intervention in LPS-activated microglia, the expression of pro-inflammatory markers is significantly downregulated, and Aβ-induced oxidative stress and neuronal apoptosis are effectively alleviated, confirming their neuroprotective effects through the regulation of neuroinflammatory pathways (Zavatti et al., 2022). Bone marrow mesenchymal stem cell-derived extracellular vesicles (BMSCs-EVs) carry miR-29c-3p, which reduces Aβ production by inhibiting BACE1 expression (Sha et al., 2021). The therapeutic mechanisms of EVs include inhibiting the overactivation of microglial cells and blocking the inflammatory damage to dopaminergic neurons. However, catalase enriched in neural stem cell-derived extracellular vesicles (NSC-EVs) in PD attenuates oxidative stress to protect dopaminergic neurons (Díaz Reyes et al., 2025). In terms of anti-inflammatory regulation, BMSCs-EVs-derived TNF-stimulated gene-6 (TSG-6) maintains dopaminergic neuronal survival by blocking the ubiquitinated degradation of LRRK2 by inhibiting the STAT3-miR-7-NEDD4 pathway, while engineered miR-7-overexpressing EVs attenuate MPP Induced neurotoxicity (Huang et al., 2022). Li et al. (2019) confirmed that BMSCs-EVs alleviate central nervous inflammation effectively by modulating immune polarization, elevating IL-10 and TGF-B, and inhibiting TNF-a and IL-12. These findings systematically uncover the core mechanisms by which stem cell EVs intervene in NDDs through multi-target, multi-pathway interactions.

### 5.2 Preclinical studies

Preclinical studies of stem cell EVs in NDDs have demonstrated therapeutic potential across a spectrum of diseases, with their mechanisms of action achieved through delivery system optimization and multi-target regulation. In the field of AD, breakthroughs in CNS-targeted delivery technology have significantly enhanced efficacy. Its molecular mechanisms involve the reduction of inflammatory factors such as IL-1β and TNF-a, the clearance of Aβ deposits, and the promotion of neuron survival and regeneration. Cui et al. (2019) developed RVG-modified MSC-EVs, which, upon intravenous injection, significantly improved cognitive function in APP/PS1 model mice and reduced levels of inflammatory factors TNF-a, IL-1β, and IL-6. Cone et al. (2021) demonstrated that intranasal administration of human bone marrow mesenchymal stem cells-derived extracellular vesicles (hBMSCs-EVs) in A5XFAD mice, which carry five familial AD mutations, significantly enhanced the clearance of Aβ plaques in the hippocampus and effectively decelerated disease progression compared to saline-treated controls. PD research reveals core pathways through multi-model validation: in the 6-OHDA injury model, BMSCs-EVs significantly repair the dopaminergic pathway through striatal-targeted delivery (Bouchez et al., 2008). Ahmed et al. (2016) found that the rotenone model further reveals that BMSCs-EVs reverse the neuroinflammatory microenvironment by regulating the TGF-β1/IL-17 axis. Nasally administered umbilical cord MSC-EVs cross the BBB and improve motor coordination in PD mice (Huang et al., 2024). Chan et al. (2023) confirmed that human adipose-derived stem cell exosomes (hADSCs-Exos) exert neuroprotective effects in PD mouse models through anti-inflammatory properties. Mechanistic studies confirm their dual role, suppressing abnormal aggregation of a-syn (Gouda et al., 2022) while upregulating tyrosine hydroxylase (TH) expression (Ahmed et al., 2016). MSC-EVs of different origins exert their effects through characteristic pathways, hADSCs-Exos target the inhibition of the IL-17 signaling axis (Ghasemi et al., 2014; Shalaby et al., 2016), and Bai et al. (2012) found that BMSCs-EVs mediate myelin repair through HGF. A systematic review by Barabadi et al. (2024) confirmed that MSC-EVs achieve improvement in neurological function scores in 80% of preclinical models. Zhang et al. (2022) reported that mouse MSC-EVs can alleviate demyelinate ion-induced functional impairment and promote neurological function recovery. In the field of ALS, ASC EVs have demonstrated neuroprotective ubiquity through dual administration routes: intravenous injection primarily improves the survival of lumbar motor neurons, while nasal administration achieves targeted enrichment in brainstem lesion areas (Kuzma-Kozakiewicz et al., 2018). Their mechanism of action involves the repair of neuromuscular junction structures and the inhibition of glial cell activation, providing new evidence for research on trans-synaptic transmission. These studies collectively build a therapeutic network of stem cell EVs from local intervention to systemic regulation, laying a theoretical foundation for clinical translation.

### 5.3 Clinical trials

The clinical translational research of stem cell EVs in NDDs has shown multidimensional breakthroughs. In the field of AD, innovative therapy through nasal delivery of MSC-EVs has achieved key progress: In a clinical trial involving patients with mild to moderate AD, treatment with MSC-EVs via nasal administration significantly improved patients’ cognitive functions. Evaluations using the Montreal Cognitive Assessment (MoCA) and Mini-Mental State Examination (MMSE) showed that scores in the treatment group improved significantly, indicating enhanced cognitive function. The daily living abilities of patients in the treatment group also improved, with enhanced capabilities in activities such as dressing and eating, suggesting that stem cell EVs therapy helps improve cognitive and self-care abilities in AD patients. Although the sample size was small and the follow-up time was short, these preliminary results provide a reference for the clinical application of stem cell EVs therapy in AD and support further large-scale trials (Xie et al., 2023; Table 1). Clinical research on PD reveals the optimization direction of EVs drug delivery systems: hypoxic preconditioning of olfactory mucosa-derived exosome (hOM-Exos) treatment significantly reduces the Unified Parkinson’s Disease Rating Scale (UPDRS) scores in PD patients, indicating improved motor function (Zhuo et al., 2024; Table 2). Research on ALS focuses on mechanism innovation and efficacy validation: although mesenchymal stem cell-derived neurotrophic factor (MSC-NTF) treatment shows changes in patients’ ALSFRS-R scores, its efficacy requires further validation (Fisher-Shoval et al., 2012; Table 3). Research on MS has achieved a leap from scale assessment to functional recovery: BMSCs-EVs treatment significantly improved scores in the 25-foot walk test (T25FW) on the EDSS scale in MS patients (Kråkenes et al., 2024). Several clinical trials have confirmed that stem cell EVs therapy can enhance patients’ motor function and quality of life, marking a paradigm shift in EVs treatment from symptom control to functional remodeling (Table 4).

**TABLE 1:** The summary of clinical trial projects on stem cell-derived exosome therapy for Alzheimer’s disease.

NCT number	Title	Status	Sponsor/collaborators	Start date	Phase
NCT06781333	Human mesenchymal stem cells (hMSC) in behavioral problems due to Alzheimer’s disease	Not yet recruiting	Bernard (Barry) Baumel, University of Miami	1/3/2025 (estimated)	Phase 2
NCT04388982	The safety and the efficacy evaluation of allogenic adipose MSC-Exos in patients with Alzheimer’s disease	Unknown status	Ruijin Hospital	1/7/2020	Phase 1 Phase 2
NCT03117738	A study to evaluate the safety and efficacy of AstroStem in treatment of Alzheimer’s disease	Completed	Nature Cell Co. Ltd.	9/5/2017	Phase 1 Phase 2
NCT06775964	Stem cell therapy for early Alzheimer’s disease	Not yet recruiting	Paul E Schulz, The University of Texas Health Science Center	2/2025 (estimated)	Phase 1 Phase 2
NCT05667649	Autologous activated adipose-derived stem cells (RB-ADSC) injected directly into the brain for mild to Moderate Alzheimer’s disease	Recruiting	Regeneration Biomedical, Inc.	14/8/2023	Phase 1
NCT02054208	Safety and exploratory efficacy study of NEUROSTEM^®^ versus placebo in patients with Alzheimer’s disease	Completed	Medipost Co. Ltd.	1/3/2014	Phase 1 Phase 2
NCT02912169	Study to assess the safety and effects of autologous adipose-derived stromal cells in patients with Alzheimer’s disease	Withdrawn	Ageless Regenerative Institute	1/11/2015	Phase 1 Phase 2
NCT01547689	Safety and efficiency of umbilical cord-derived mesenchymal stem cells (UC-MSC) in patients with Alzheimer’s disease	Unknown status	Affiliated Hospital to Academy of Military Medical Sciences	1/3/2012	Phase 1 Phase 2
NCT02672306	Safety and exploratory efficacy study of UCMSCs in patients with Alzheimer’s disease	Unknown status	South China Research Center for Stem Cell and Regenerative Medicine	20/10/2017	Phase 1 Phase 2
NCT01297218	The safety and the efficacy evaluation of NEUROSTEM^®^-AD in patients with Alzheimer’s disease	Completed	Medipost Co. Ltd.	1/2/2011	Phase 1

**TABLE 2:** The summary of clinical trial projects on stem cell-derived exosome therapy for Parkinson’s disease.

NCT number	Title	Status	Sponsor/collaborators	Start date	Phase
NCT06687837	Treating Parkinson’s disease through transplantation of autologous stem cell-derived dopaminergic neurons	Recruiting	Jeffrey S. Schweitzer, MD, Ph.D.	1/12/2024	Phase 1
NCT06482268	Transplantation of human iPS cell-derived dopaminergic progenitors (CT1-DAP001) for PD.	Recruiting	University of California, San Diego	1/6/2024	Phase 1
NCT06145711	A clinical trial of Parkinson’s disease treatment by HiPSCs derived dopaminergic neural precursor cells	Recruiting	Shanghai East Hospital	23/11/2023	Not applicable
NCT06141317	Randomized clinical trial in Parkinson’s disease patients using pluripotent adipose stem cells	Active, not recruiting	ClusterXStem-Costa Rica	23/6/2023	Phase 1 Phase 2
NCT05901818	Safety and efficacy of autologous iNSC-DAP in the treatment of Parkinson’s disease	Recruiting	Xuanwu Hospital, Beijing	13/6/2023	Phase 1
NCT05887466	Study to evaluate the safety and efficacy of ESC-derived dopamine progenitor cell therapy in PD patients	Active, not recruiting	S. Biomedics Co., Ltd.	9/5/2023	Phase 1 Phase 2
NCT05691114	Precise transplantation of human amniotic epithelial stem cells into lateral ventricle for Parkinson’s disease	Recruiting	Shanghai East Hospital	1/2/2023	Phase 1
NCT05635409	A trial to determine the safety and tolerability of transplanted stem cell derived dopamine neurons to the brains of individuals with Parkinson’s disease	Active, not recruiting	Region Skane	30/11/2022	Phase 1
NCT05152394	Safety of cultured allogeneic adult umbilical cord derived mesenchymal stem cells for Parkinson’s disease	Not yet recruiting	The Foundation for Orthopaedics and Regenerative Medicine	1/1/2022	Phase 1
NCT04414813	Stereotactic transplantation of hAESCs for Parkinson’s disease	Completed	Shanghai East Hospital	8/10/2020	Early phase 1
NCT03684122	Use of mesenchymal stem cells (MSCs) differentiated into neural stem cells (NSCs) in people with PD	Unknown status	University of Jordan	1/6/2018	Phase 1 Phase 2
NCT03119636	Safety and efficacy study of human ESC-derived neural precursor cells in the treatment of Parkinson’s disease	Unknown status	Chinese Academy of Sciences	1/5/2017	Phase 1 Phase 2
NCT01446614	Mesenchymal stem cells transplantation to patients with Parkinson’s disease	Unknown status	Guangzhou General Hospital of Guangzhou Military Command	1/10/2011	Phase 1 Phase 2

**TABLE 3:** The summary of clinical trial projects on stem cell-derived exosome therapy for amyotrophic lateral sclerosis.

NCT number	Title	Status	Sponsor/collaborators	Start date	Phase
NCT02492516	Intravenous injection of adipose derived mesenchymal stem cell for ALS	Completed	Royan Institute	9/2014	Phase 1
NCT01348451	Human spinal cord derived neural stem cell transplantation for the treatment of amyotrophic lateral sclerosis	Unknown status	Neuralstem Inc.	1/2009	Phase 1
NCT01254539	Clinical trial on the use of autologous bone marrow stem cells in amyotrophic lateral sclerosis	Completed	Fundacion para la Formacion e Investigacion Sanitarias de la Region de Murcia	10/2010	Phase 1 Phase 2
NCT01494480	The clinical trial on the use of umbilical cord mesenchymal stem cells in amyotrophic lateral sclerosis	Unknown status	General Hospital of Chinese Armed Police Forces	3/2012	Phase 2
NCT00855400	Clinical trial on the use of autologous bone marrow stem cells in amyotrophic lateral sclerosis	Completed	Fundacion para la Formacion e Investigacion Sanitarias de la Region de Murcia	2/2007	Phase 1 Phase 2
NCT01640067	Human neural stem cell transplantation in amyotropahic lateral sclerosis	Completed	Angelo Luigi Vescovi, Azienda Ospedaliera Santa Maria	12/2011	Phase 1
NCT01933321	Effect of intrathecal administration of hematopoietic stem cells in patients with amyotrophic lateral sclerosis	Completed	David Gomez Almaguer, Hospital Universitario Dr. Jose E. Gonzalez	9/2012	Phase 2 Phase 3
NCT05003921	Safety of cultured allogeneic adult umbilical cord derived mesenchymal stem cell intrathecal injection for ALS	Suspended	The Foundation for Orthopaedics and Regenerative Medicine	12/2022	Phase 1
NCT01142856	Mesenchymal stem cells for treatment of amyotrophic lateral sclerosis	Completed	Mayo Clinic	6/2010	Phase 1
NCT06344260	Neural stem cell treatment for amyotrophic lateral sclerosis	Recruiting	Casa Sollievo della Sofferenza IRCCS	25/1/2024	Phase 2

**TABLE 4:** The summary of clinical trial projects on stem cell-derived exosome therapy for multiple sclerosis.

NCT number	Title	Status	Sponsor/collaborators	Start date	Phase
NCT05532943	Evaluate the safety and efficacy of allogeneic umbilical cord mesenchymal stem cells in patients with multiple sclerosis	Recruiting	Ever Supreme Bio Technology Co., Ltd.	8/9/2023	Phase 1 Phase 2
NCT05003388	Safety of cultured allogeneic adult umbilical cord derived mesenchymal stem cell intravenous infusion for MS	Recruiting	The Foundation for Orthopaedics and Regenerative Medicine	26/6/2021	Phase 1
NCT02239393	Safety and efficacy of intravenous autologous mesenchymal stem cells for MS: a phase 2 proof of concept study	Completed	Ottawa Hospital Research Institute	6/2015	Phase 2
NCT06360861	Evaluate the safety and feasibility of allogeneic mesenchymal stem cells in patients with multiple sclerosis	Completed	Tehran University of Medical Sciences	23/7/2019	Phase 1
NCT00395200	Mesenchymal stem cells in multiple sclerosis	Completed	Peter Connick, University of Cambridge	7/2008	Phase 1 Phase 2
NCT04823000	Effects of repeated mesenchymal stem cells (MSC) in patients with progressive multiple sclerosis	Completed	Hadassah Medical Organization	1/1/2013	Phase 1 Phase 2
NCT01730547	Mesenchymal stem cells for multiple sclerosis	Completed	Lou Brundin, Karolinska Institutet	2/2013	Phase 1 Phase 2
NCT04749667	Study of mesenchymal autologous stem cells as regenerative treatment for multiple sclerosis	Active, not recruiting	Haukeland University Hospital	9/8/2021	Phase 1 Phase 2
NCT01854957	Mesenchymal stem cells for multiple sclerosis	Unknown status	Antonio Uccelli, University of Genova	7/2012	Phase 1 Phase 2
NCT02166021	Clinical efficacy of autologous mesenchymal bone marrow stem cells in active & progressive multiple sclerosis	Completed	Dimitrios Karussis, Hadassah Medical Organization	29/1/2015	Phase 2

## 6 Advantages and challenges

Stem cell EVs have significant advantages in the treatment of NDDs (Ranganath et al., 2012; Sajeesh et al., 2020; Santamaria et al., 2021). The lipid bilayer membrane of EVs effectively shields cargo components (miRNAs, enzymes) from degradation by blood-borne nucleases and proteases, thereby prolonging their circulatory half-life. In contrast, free anti-inflammatory factors like IL-10 undergo rapid clearance in systemic circulation. EVs demonstrate BBB penetration capability through receptor-mediated transcytosis (Saint-Pol et al., 2020; van Niel et al., 2018), whereas most anti-inflammatory proteins (including antibodies) lack direct access to the CNS. Notably, EVs can detect inflammatory signals in pathological tissues via surface molecules such as integrins, and exhibit tropism toward injury sites through biological recognition mechanisms (Shimaoka et al., 2019). This active targeting capacity (Liu et al., 2022b) remains unattainable for free molecular therapeutics. In terms of immune regulation, MSC-EVs can function through multiple mechanisms: they achieve bidirectional immune modulation by regulating the TLR/NF-κB signaling pathway and the Th17/Treg cell balance (de Godoy et al., 2018; Wang J. et al., 2020), while simultaneously delivering neurotrophic factors to promote neuron survival and reduce apoptosis rates (Fakouri et al., 2024; Namini et al., 2023; Tambe et al., 2024). Studies have also found that EVs can activate neural regeneration-related transcription factors such as Sox2/Pax6. Engineered EVs modified via the Notch signaling pathway can significantly enhance the differentiation efficiency of oligodendrocytes (Fričová et al., 2020). Thanks to their low immunogenicity (Heris et al., 2022) and potential for large-scale production (Chan et al., 2023), these characteristics make them a promising vehicle for clinical translation in neural repair.

However, the clinical application of this technology still faces significant challenges. Firstly, cross-contamination often occurs during EVs isolation and purification, and existing techniques are unable to completely remove other cellular components, which directly affects the purity and functional stability of the preparation (Moon et al., 2023). Secondly, although research indicates that EVs can target brain lesion areas via the BBB, their non-specific distribution in systemic circulation may lead to off-target effects (Wan et al., 2020). Intravenous injection acts quickly but is prone to off-target effects and is suitable for systemic diseases (Wiklander et al., 2015). Intraperitoneal targeting affects the metabolic system (Zhou et al., 2020), oral administration targets the digestive system (Umezu et al., 2021), and localized injections are precise and efficient but require caution regarding abnormal accumulation (Wang D. et al., 2022). Therefore, the choice should be based on therapeutic goals and targeting the specific organs needed (Song et al., 2022). Although the surface protein CD47 sends a “don’t eat me” signal by binding to SIRPa on macrophages, thus reducing the binding of EVs to macrophages and maximizing circulation time (Koh et al., 2017), optimizing targeting remains a challenge. Furthermore, the complex composition of EVs contents and their interaction mechanisms with host cells are not yet fully elucidated, which poses obstacles to the design and standardization of treatment protocols (Fričová et al., 2020). In order to solve the current obstacles based on stem cell EVs treatment, there is still a lack of uniform standards and guidelines for the preparation, storage and application of EVs. This lack of standardization may lead to inconsistent results between different studies, thus affecting the clinical application of EVs, and its potential mechanism needs further study.

## 7 Conclusion

Stem cell EVs demonstrate immense potential in the treatment of NDDs due to their ability to cross the BBB and multifunctional regulatory properties. Current research confirms that EVs alleviate neuroinflammation through mechanisms including immune response modulation, oxidative stress reduction, neuroprotection enhancement, and cellular metabolism optimization. These findings not only provide novel insights into the complexity of neuroinflammation but also establish a foundation for innovative therapeutic strategies. Longitudinal monitoring of inflammatory factor profiles within EVs isolated from patient serum or cerebrospinal fluid could enable the development of novel therapeutic biomarkers, facilitating early efficacy prediction and dynamic intervention. Emerging studies propose synergistic integration of EVs-based therapies with existing pharmacological agents to amplify therapeutic outcomes. Crucially, standardization of EVs isolation and characterization methodologies is essential for ensuring treatment consistency. Researchers are currently engineering MSCs via gene editing to optimize exosomal cargo composition, while developing advanced biomaterials to enhance targeting specificity, innovations poised to overcome current technical limitations. The anti-inflammatory properties of MSC-EVs hold significant promise for NDD management. Future investigations should prioritize mechanistic elucidation of EVs-mediated neuroinflammatory regulation, neuronal protection, and regeneration, with emphasis on identifying precise molecular targets and signaling pathways. Exploratory integration with emerging technologies such as brain-computer interfaces and cerebral organoid transplantation may enable neural network remodeling. Multidimensional innovations in this field are anticipated to transcend conventional therapeutic boundaries, ultimately driving a paradigm shift from delaying disease progression to achieving functional cure in neurodegenerative disorders.
